# Tuberculosis service disruptions and adaptations during the first year of the COVID-19 pandemic in the private health sector of two urban settings in Nigeria—A mixed methods study

**DOI:** 10.1371/journal.pgph.0001618

**Published:** 2023-03-24

**Authors:** Charity Oga-Omenka, Angelina Sassi, Nathaly Aguilera Vasquez, Elaine Baruwa, Lauren Rosapep, Benjamin Daniels, Bolanle Olusola-Faleye, Lavanya Huria, Abdu Adamu, Benjamin Johns, Jishnu Das, Madhukar Pai

**Affiliations:** 1 School of Public Health Sciences, University of Waterloo, Waterloo, Ontario, Canada; 2 McGill International TB Centre, Montreal, Canada; 3 Department of Epidemiology, Biostatistics and Occupational Health, McGill University, Montreal, Canada; 4 Sustaining Health Outcomes through the Private Sector (SHOPS) Plus/Abt Associates, Lagos, Nigeria; 5 School of Public Policy, Georgetown University, Washington, DC, United States of America; Makerere University, UGANDA

## Abstract

Nigeria has the second largest share of undiagnosed TB cases in the world and a large private health sector estimated to be the point of initial care-seeking for 67% of TB patients. There is evidence that COVID-19 restrictions disrupted private healthcare provision, but insufficient data on how private healthcare provision changed as a result of the pandemic. We conducted qualitative interviews and a survey to assess the impact of the pandemic, and government response on private healthcare provision, and the disruptions providers experienced, particularly for TB services. Using mixed methods, we targeted policymakers, and a network of clinical facilities, laboratories, community pharmacies, and medicine vendors in Kano and Lagos, Nigeria. We interviewed 11 policymakers, surveyed participants in 2,412 private facilities. Most (n = 1,676, 70%) facilities remained open during the initial lockdown period, and most (n = 1,667, 69%) offered TB screening. TB notifications dipped during the lockdown periods but quickly recovered. Clinical facilities reported disruptions in availability of medical supplies, staff, required renovations, patient volume and income. Few private providers (n = 119, 11% in Kano; n = 323, 25% in Lagos) offered any COVID-19 screening up to the time of the survey, as these were only available in designated facilities. These findings aligned with the interviews as policymakers reported a gradual return to pre-COVID services after initial disruptions and diversion of resources to the pandemic response. Our results show that COVID-19 and control measures had a temporary impact on private sector TB care. Although some facilities saw decreases in TB notifications, private facilities continued to provide care for individuals with TB who otherwise might have been unable to seek care in the public sector. Our findings highlight resilience in the private sector as they recovered fairly quickly from pandemic-related disruptions, and the important role private providers can play in supporting TB control efforts.

## Background

Prior to COVID-19, an estimated 3–4 million TB cases went undetected or unreported to national TB programs every year [1]. In Nigeria, the focus of this study, the proportion of undetected TB cases was estimated to be 70% in 2019 [1]. During the COVID-19 pandemic, there were reports that lockdowns, movement restrictions, and fears of acquiring COVID-19 at health facilities greatly limited patient health-seeking which may have hampered TB diagnosis and service provision and access [2,3]. The combination of non-pharmaceutical interventions such as lockdowns and the shifting of resources to COVID-19 has contributed to a sharp decline globally of 18% in the number of diagnosed TB cases in 2020 compared to 2019 [4,5]. This could result in excess mortality of half a million and represents a significant setback in TB control [2].

Nigeria has had the highest number of estimated TB cases of any African country since 2018 [6,7], and currently has the second largest share of undiagnosed TB cases in the world according to WHO estimates. Up to 67% of initial care-seeking in Nigeria occurs in the private sector [8,9], yet the private sector has historically accounted for a small proportion of TB notifications, making Nigeria an important setting for interventions to strengthen public-private partnerships [9,10]. Due to a push for initiatives that focus on public-private mix (PPM), the proportion of total notifications contributed by the private sector in Nigeria increased from 8% in 2012 to 26% in 2020 [9,11], thereby helping to reduce gaps in TB notification and underlining the importance of multisectoral efforts to end TB [12].

Despite this progress, TB diagnosis and notification in the private sector may have regressed overall as a result of COVID-19. Following the index case on February 27^th^ 2020, the Nigerian Government issued a series of stay-at-home order and cessation of non-essential movement and activities beginning on March 30^th^ in Lagos, Ogun, the Federal Capital Territory (FCT) and later in Kano state, to curb the spread of COVID-19 [13]. After five weeks of a federally mandated lockdown, restrictions were gradually eased. COVID-19 infection control measures were implemented early in the pandemic to reduce infection and disease transmission; however, earlier reports indicated that they also led to challenges in accessing health services, especially for already vulnerable populations such as individuals with TB in Nigeria [6,14,15], as they did in other high burden countries [16,17].

As of December 2022, there were over 266,000 confirmed COVID-19 cases and 3,000 COVID-related deaths in Nigeria [18], although these numbers were likely underestimated [19]. In the early days of the pandemic, only public facilities in Nigeria were allowed to treat COVID-19 patients, and screening capacity was limited in the early months of the pandemic [13]. In July 2020, the Federal Ministry of Health announced that COVID-19 sample collection would be scaled up to all eligible public and private hospitals [20]. While it is clear that both the reaction to the pandemic and the shifting of resources to COVID-19 could have impacted TB services, to date, few studies have been published on the impact of COVID-19 on the availability and delivery of TB services in the private sector.

In Nigeria, unlike most other high burden countries, overall TB case notifications increased (by 16%) in 2020 compared to 2019 [2], due in part to concerted efforts by the TB program to increase and integrate TB active case finding into COVID-19 sensitization in all states [21]. The 2021 Global TB report highlighted Nigeria as an exception to the rule with regards to the global impact of COVID-19 on TB services due to the fairly rapid recovery after initial disruptions [2]. However, notification and treatment coverage for drug-resistant TB, which were among the lowest in the world in previous years, decreased by 14% and 20% respectively, after the pandemic hit [2].

Here, we aimed to assess the status of TB service provision in the private sector, any changes, service disruptions and adaptations to the COVID-19 pandemic, particularly regarding provision of TB services. Our research questions were: 1) What proportion of private providers report closures, and for how long, during the first year of the COVID-19 pandemic. 2) Were there changes to availability and costs of TB testing and treatment services? 3) How did private providers adapt to Government pandemic control measures? 4) Were there significant differences between States in service delivery and adaptations due to the pandemic?

## Methods

### Study design and background of COVET study

This paper uses data from the COVID Effects on TB Services in the Private Sector (COVET) study that aimed to evaluate the impact of the COVID-19 pandemic on the private healthcare sector in India, Indonesia, and Nigeria. Our concurrent mixed methods study was carried out between May and July 2021. We interviewed policymakers and surveyed private providers after the second COVID-19 wave in Kano and Lagos. The respondents included federal and state policymakers, medicine vendors, community pharmacies, primary care facilities and laboratories.

Our policymaker interviews focused on COVID-related regulations as well as the perceived impact on private providers. The facility and provider survey covered the provision of TB services. We surveyed private sector providers in Kano and Lagos States, whose combined population accounts for over 13% of Nigeria’s 206 million estimated population in 2020 [22,23]. These providers were sampled from the Sustaining Health Outcomes through the Private Sector (SHOPS Plus) Program, led by Abt Associates. As part of its program mandate, SHOPS Plus had previously formed networks of private health service providers including clinical facilities, stand-alone laboratories, community pharmacies (CPs), and medicine vendors, who are trained and systematically engaged to provide appropriate TB screening, diagnosis, and treatment practices [24]. The SHOPS Plus program activities were implemented in Kano and Lagos, two of the states with the highest TB burden and numbers of private sector providers in Nigeria, as part of efforts to scale up private sector involvement in TB. We also reviewed routine program data on TB private sector service provision between May 2018 and June 2021, which includes the timings for the first two waves of the COVID-19 pandemic in Nigeria—April 2020 to November 2020 and November 2020 to May 2021. Using these data, we describe changes in TB service provision during these two COVID-19 waves and identify challenges and adaptations made by private providers in the first year of the pandemic.

### Sampling

We conducted key informant interviews with policymakers and stakeholders working at the state and national levels based on their positions and potential to be knowledgeable about the country’s response to COVID and TB.

We also collected survey data in Kano and Lagos, Nigeria. Providers within the SHOPS Plus Program were interviewed. Additional providers not supported by the SHOPS Plus network were also sampled in this study to ensure that the sample had a broad representation of facilities in Kano and Lagos (S1 Fig). An additional module of the survey was administered to only clinical facilities for an understanding of the efforts put into place to address both COVID-19 and TB, as well as the impact of regulatory changes and pandemic-related disruptions on clinical facilities.

As of June 2021, Nigeria’s National Health Facility Registry indicated that there were approximately 302 private clinics and hospitals operating in Kano and 1,875 in Lagos [25]. The Nigeria Health Facility Register (NHFR) reports a total of 1,476 and 2,333 clinical facilities in Kano and Lagos respectively, with private facilities representing 14% and 80% of these.

The enumeration list used two different datasets: the SHOPS Plus monthly program monitoring dataset (service delivery data submitted by network facilities monthly between April 2018 and September 2020), and a list of facilities that were surveyed in 2018 to gauge interest and suitability to participate in a SHOPS Plus program network. The setup of the SHOPS Plus network is detailed in a prior publication [24]. This survey focused on collecting data on the impact of COVID-19 from private facilities providing TB services within the SHOPS Plus network, and from a few non-network private facilities (from the 2018 Assessment dataset excluded from the SHOPS program list). The enumeration list for this study consisted of a total of 2,926 facilities, including 207 clinical facilities in Kano (69% of the total number of clinical facilities listed in the national register for Kano) and 419 clinical facilities in Lagos (or 22% of the total for Lagos) (S1 Fig). Exclusions included permanent closures before or after the COVID-19 waves (11.4% of unique facilities that had ever reported data as a SHOPS Plus network member) and facilities with no location information (9.7%) (S1 Fig).

### Data collection

#### Qualitative data collection

We interviewed the 11 policymakers and stakeholders between May and July 2021, after asking and receiving their consent. At the state level, we interviewed a total of 8 senior officials each at the Kano (4) and Lagos (4) States Ministries of Health, private health facility governing bodies. At the national level, we interviewed senior managers within the National TB program. We asked policymakers about their role in TB coordination in the private sector, any new policies or regulations and enforcement, the impact of COVID on the private sector, as well as the response and performance of the private sector.

Interviews were between 30 and 45 minutes and were conducted in English and recorded via WebEx by trained field officers engaged by Abt Associates. Interview recordings were transcribed.

#### Quantitative data collection

Between May 10^th^ and June 11^th^, 2021, data were collected using a cross-sectional survey containing two modules—a general and an extended COVID module. The general module, containing 68 questions was administered in person to the facility manager within all consenting facilities (n = 2,412) and included questions on facility closures since the pandemic began, changes in outpatient (OPD) visits, costs of services, TB service availability, and telemedicine use. The extended COVID module, administered to 628 clinical facilities, contained 26 additional questions on general provider characteristics, how private providers were affected by the lockdowns, and what adaptations and changes they made to recover. In addition, this survey also inquired about service disruptions during the COVID-19 pandemic and new protocols that were adopted, for instance, around personal protective equipment (PPE), limits on the number of clients and online consults. Data were also collected on occurrence, management, and treatment of TB cases at the facility.

Before the survey was carried out, the team held sensitization meetings with officials of the Kano and Lagos State Ministries of Health (MOH). We translated the survey questionnaire into Hausa, and then back translated to English to avoid distortion and maintain the integrity of the questions. The questionnaire was also pretested, piloted, and updated based on field experience. We programmed the survey questionnaire into SurveyCTO and trained field officers in both locations over a five-day period for each survey. Only personnel who performed satisfactorily on the training evaluation were deployed.

Prior to survey commencement, data collectors obtained verbal informed consent from the respondents. Before each interview, participants were asked to choose the language (English or Hausa) that they wanted to complete the survey in.

### Data analysis

Interview transcripts were thematically coded using the Quirkos software and Microsoft Excel. Survey data were analyzed using R Studio (RStudio, version 1.4.1106), Stata (version 16.1, StataCorp LLC, College Station, TX), and Tableau version 2021.2. Descriptive statistics were generated including frequencies and percentages. Two-proportion Z-tests were used to determine significant differences between proportions.

### Ethical considerations

This study received ethical approval from the Research Institute of the McGill University Health Centre (RI-MUHC) ethics board, the Abt Associates Institutional Review Board (IRB) and the Health Research Ethics Committee (HREC) in the two Nigerian states: HREC Kano State MOH and HREC Lagos State University Teaching Hospital (LASUTH). Data collectors obtained verbal informed consent from respondents prior to the commencement of interviews. The data collectors also assured the respondents of the confidentiality and anonymity of the responses provided during data collection. Further, respondents were made to understand that their participation is voluntary, and they could withdraw from or discontinue the interview at any time.

## Results

### Characteristics of respondent and non-respondent facilities

**Fig 1:**
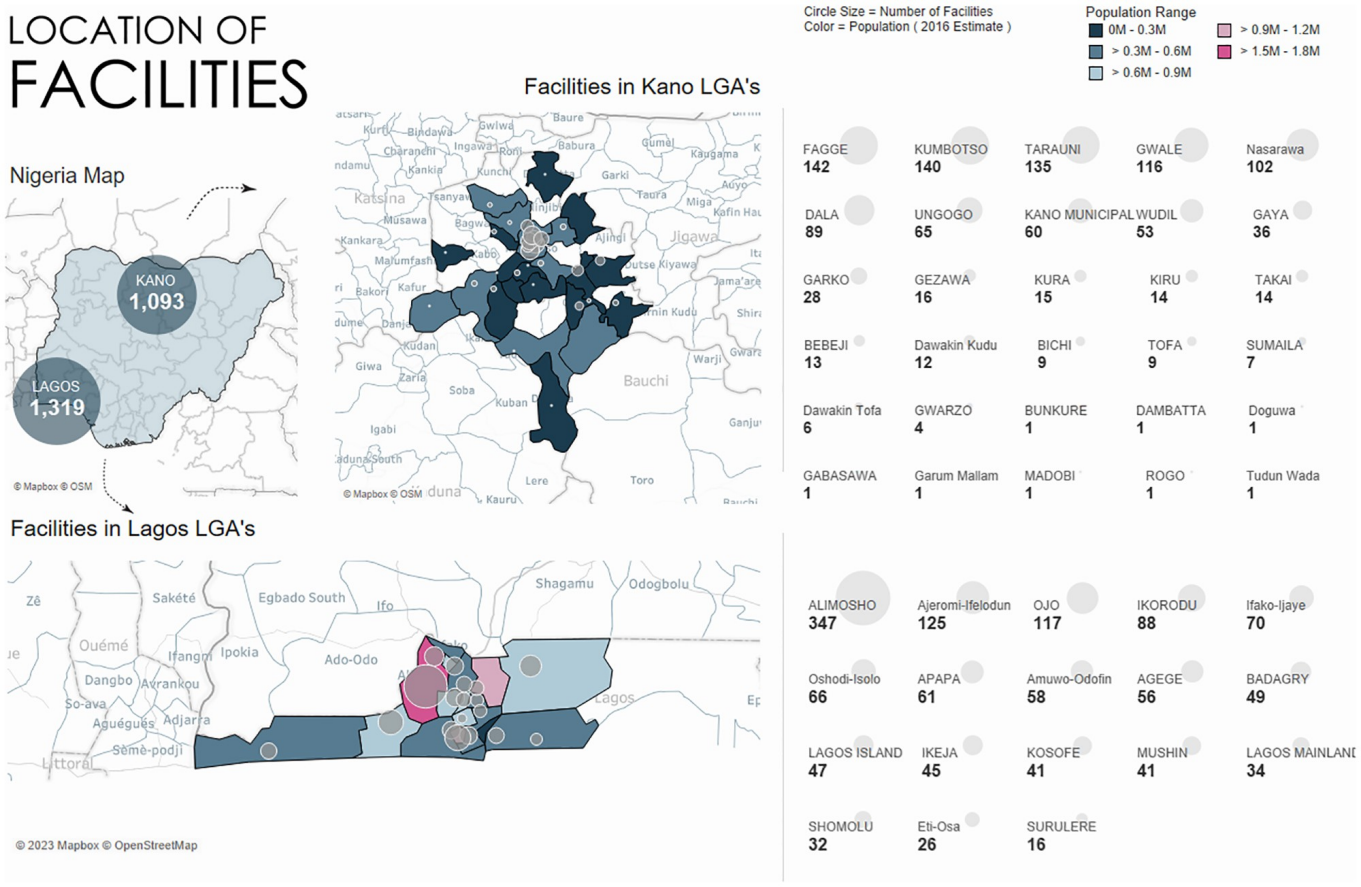
Location of facilities in Kano and Lagos.

A total of 2,412 out of 2,926 facilities in the survey enumeration list were successfully visited and consented to be surveyed (82.4%) (S1 Fig). The facility survey successfully located 1,093 and 1,319 facilities in Kano and Lagos, respectively (Fig 1).

The facilities surveyed were predominantly within the SHOPS Plus network (88%) (Table 1). A comparison of respondent and non-respondent facilities is shown in Table 1. The proportions of non-response facilities were lower in Kano compared to Lagos, non-network facilities compared to networked facilities, and community pharmacies compared to other facility types.

**Table 1:** Comparison of respondent and non-respondent facilities.

Characteristic	Response Status		Overall, N = 2,926 n(%)
	Response, N = 2,412 n (%)	Non-response, N = 514 n (%)	
**State**			
Kano	1,093 (84%)	207 (16%)	1,300 (100%)
Lagos	1,319 (81%)	307 (19%)	1,626 (100%)
**Network status**			
SHOPS Plus	1,659 (88%)	237 (13%)	1,896 (100%)
Non-network	730 (72%)	277 (28%)	1,007 (100%)
Backup list	23 (100%)	0 (0%)	23(100%)
**Facility Designation**			
Private-for-profit	2,381 (100%)	0 (0%)	2,381 (100%)
Faith-based organizations (FBO)	26 (100%)	0 (0%)	26 (100%)
Non-Governmental Organization (NGO)	5 (100%)	0 (0%)	5 (100%)
**Facility type**			
Medicine vendors	1,183 (81%)	275 (19%)	1,458 (100%)
Clinical Facility	628 (90%)	73 (10%)	701 (100%)
Community Pharmacy	343 (75%)	114 (25%)	457 (100%)
Laboratories	258 (83%)	52 (17%)	310 (100%)
**Accreditation**			
Yes	2,145 (100%)	0 (0%)	2,145 (100%)
No	267 (100%)	0 (0%)	267 (100%)

### Characteristics of surveyed facilities

Of the 2,412 surveyed facilities, 55% (1,319) were in Lagos, 69% (1,659) were SHOPS Plus facilities, and 99% (2,381) were private for-profit facilities. The facility types included medicine vendors (1,183 or 49%), clinical facilities (628 or 26%), community pharmacies (343 or 14%) and laboratories (258 or 11%). A total of 2,145 facilities (89%) were accredited by the State Ministry of Health, healthcare facilities accreditation and professional regulating authorities.

### Service disruptions during the COVID-19 pandemic waves

**Fig 2:**
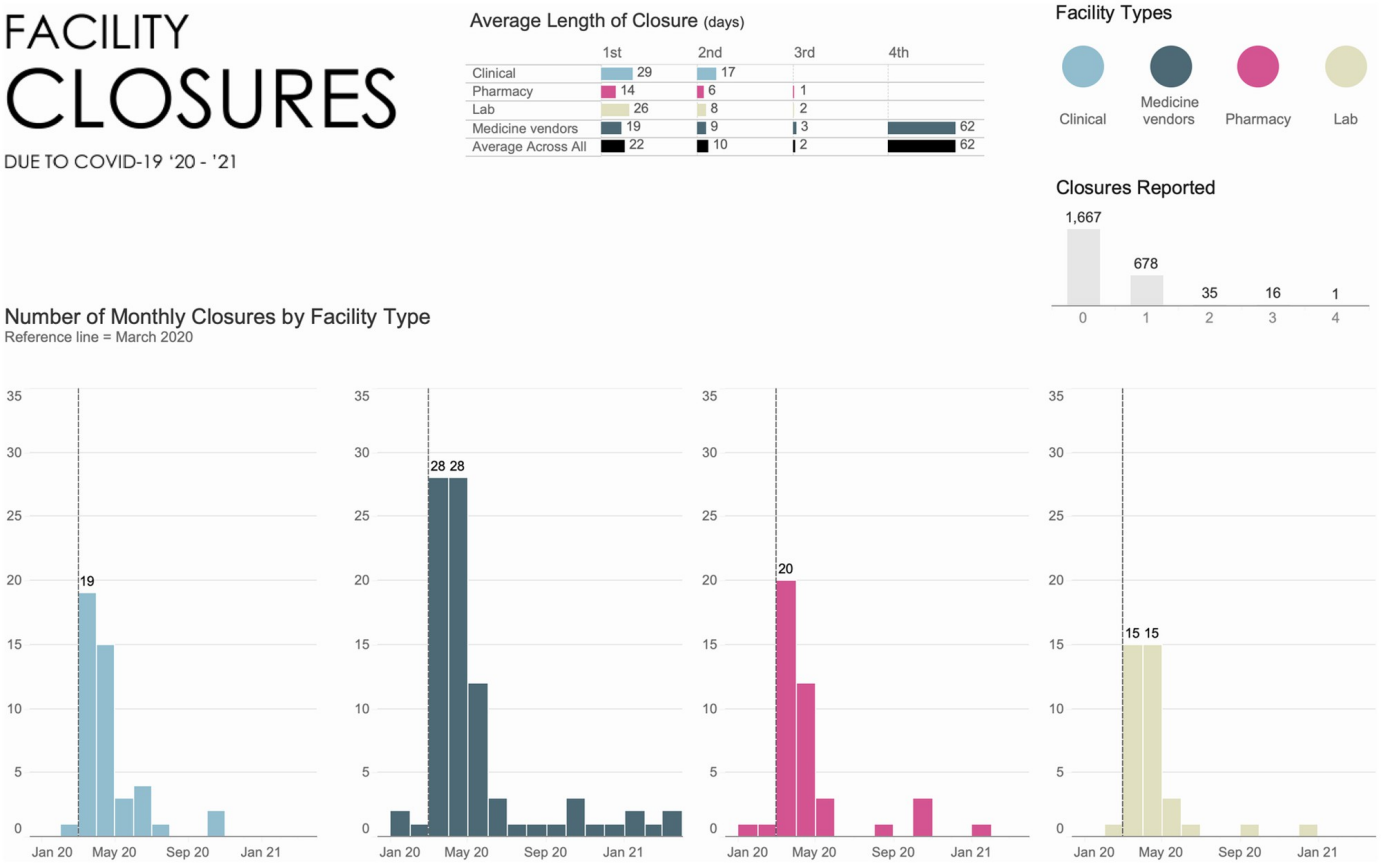
Facility closures due to COVID-19, 2020–2021.

The majority (70%, n = 1,667) of facilities reported that they remained open throughout the lockdown period, 28% facilities reported closing once (n = 687) and 2% closed more than once (n = 49). Medicine vendors were most likely to close more than once (Fig 2), but most were reopened by July 2020, the peak of the first wave. Medicine vendors also reported closures for the longest periods of time overall, even though clinical facilities had longer lengths of closures during the initial lock down periods. Frequency and duration of closures were lower among pharmacies and medicine vendors than in laboratories and clinical facilities. Of those facilities that closed once, the most common reason given was government mandates or lockdowns (684 facilities, or 93%). Most closures were temporary, beginning mostly right after the March 2020 lockdown and sometimes lasting up to June 2020, with wide variations in lengths of closures. Very few facilities (0.5%, n = 13) reported any closures after September 2020, reflecting the fact that there were no official mandates requiring closures past 2020 in Nigeria.

Interviews with policymakers indicated some disruptions in essential services, with many TB patients unable to access treatment according to schedule due to lockdown restrictions in the first COVID wave (e.g. March/April 2020), even though a majority of participating providers said their facilities were open throughout. Almost all key informants noted that patients avoided visiting health facilities (especially public facilities) due to the fear of being diagnosed with COVID-19. However, many interviewed, including senior national TB program (NTP) managers, indicated that since the first wave of COVID, services had since returned to normal.

*“There are so many other essential services that are affected during COVID-19, all attention now drawn to COVID-19, the issue of maternal and child health, reproductive health is affected, people with TB were affected, everybody is focusing on COVID-19 and then all those essential services, immunization, childhood immunization were affected and indirectly that had affected, you that BCG, the vaccine for childhood prevention of tuberculosis.”*

Lagos State Public Health Official.

*“Things … have reverted to normal… Because, a number of engagements, dialogues [were] put into place and you know it is an issue of global health, and we have development agencies such as SHOP Plus and we have funders like the World Bank, and … BMGF and other global financing facilities are coming … to do the needful and also now collaborating with the State government.”*

Senior MOH Staff

However, NTP managers differed from state TB staff and the survey data in their overall perception of how widespread the facility shutdowns were. While the NTP managers interviewed mostly perceived private sector shutdowns to be temporary and not so widespread, state staff shared that there were periods of near total shutdowns of facilities.

*“From the data we have and the data we analyzed; we saw more [closures in] the public sector. In fact, presently, we’re not aware of any private sector facility because we found that in the public sector, when the healthcare workers became apprehensive … or [are] infected… there’s a vacuum or disruption in service. But in the private sector, it’s more like there’s always someone, you’ll see easy task shifting within the private sector. So presently, I’m not aware of any of the private sector [closures].”*

NTP M&E staff.

*“I do not know of any [private facility closures], but it is very plausible.”*

Senior NTP manager.

*“So, particularly for TB program, … there are over 500 … private health facilities … more than 60% or 70% of them at one point were shut down. In fact, it was a total shut down for a period of 2 weeks. At one point, … the first wave when we first had the disease here in Kano … it was really a serious problem and that was the time when the Executive Governor had [brought out the lockdown] policy to be enforced”*

Senior MOH Staff, Kano State

*“… private facilities in Lagos… most facilities were shut down during the early… that was in April 2020, in the first phase of the pandemic where most facilities were shut down because of the COVID but they have obviously reopened especially the private and also … the public facilities.*

Senior TB Manager, Lagos State

### TB notification trends before and after the COVID-19 pandemic

**Fig 3:**
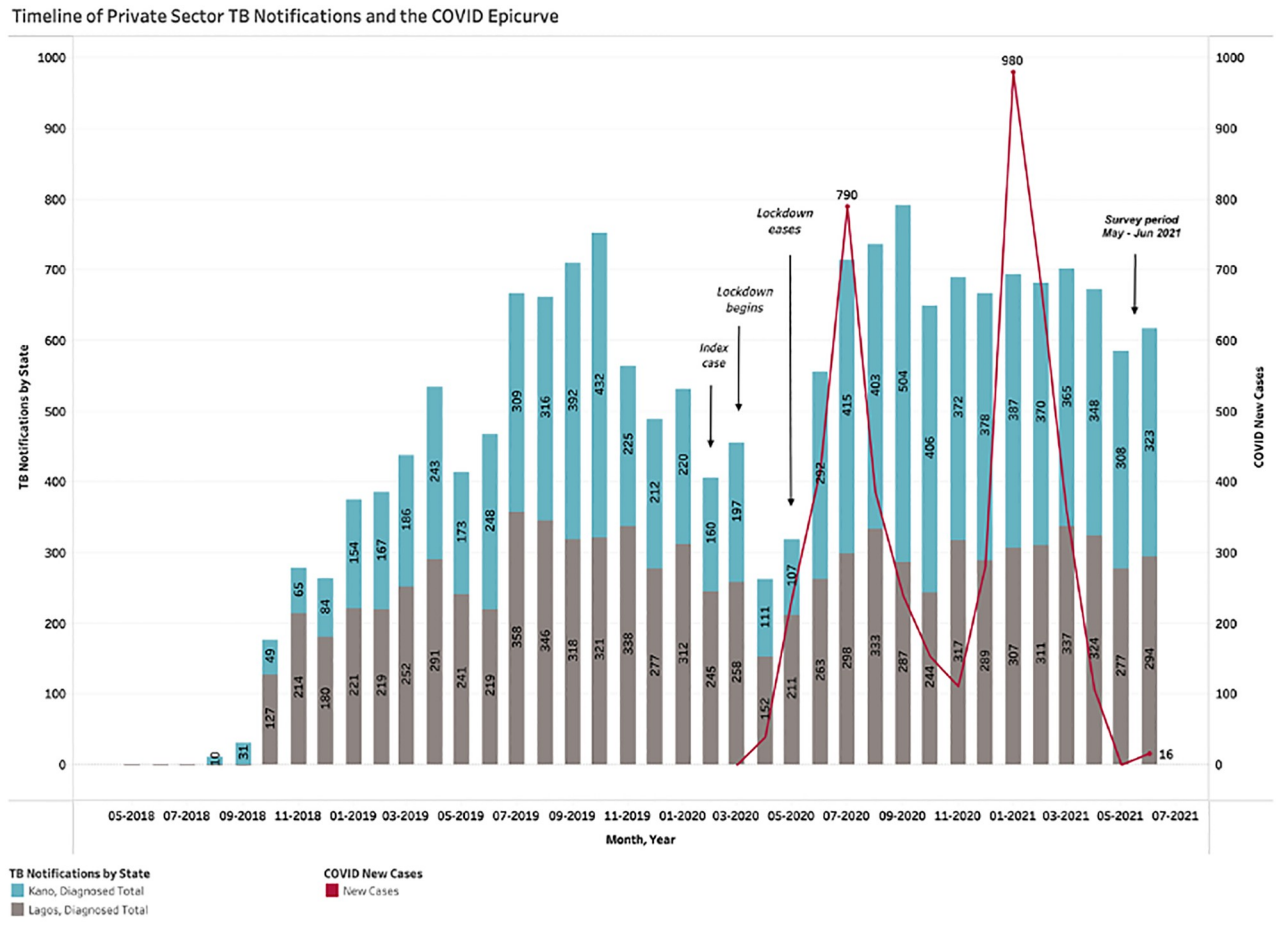
COVID epi curve and TB notifications in Kano and Lagos from January 2019 to July 2021.

The trend in TB service provision within SHOPS Plus network spanning from January 2019 to July 2021 are shown in Fig 3. Overall, TB notifications in both states were unaffected by increases in COVID-19 cases. In the 2 states, total SHOPS Plus notifications dropped dramatically in March 2020 coinciding with the beginning of the lockdown measures and begin to recover in April with the easing of lockdown measures. The black line running down the chart in Fig 3 represents the onset of the COVID-19 lockdowns in March 2020. In both Kano and Lagos, although Outpatient (OPD) attendance, total screened for TB, total presumptive TB and total diagnosed with TB show trends dipping during or immediately following March 2020, numbers swiftly recovered and returned to pre-pandemic levels in the following months. Total OPD attendance were comparatively higher in Lagos, while total presumptive numbers were higher in Kano.

Key informants from the National TB program were all in agreement that the private sector was able to weather the shock of COVID-19, by being better-positioned to serve patients during this time. This is in part because public facilities became the locus of the COVID response, leading to some health worker absenteeism and patient avoidance of public facilities (due to fear of contracting COVID). As a result, the national program observed an increase in private sector notifications.

*“It is important to note that [the early days of the pandemic] came with a lot of apprehension, panic, fear, because it [was]a new virus and most people were still not sure of the mode of transmission… There was apprehension, … fear, even among the healthcare providers … So, it impacted negatively the healthcare delivery … Most people avoided the public health facilities because they were scared of going to the hospital. Doctors …, healthcare workers were apprehensive; … every person that presented with cough, fever were taken … a probable case of COVID-19, and so people were… forced to seek help at the private health facilities, [giving way] to … increment of attendance to private hospitals and … within that time, the TB notification was relatively higher in the private health facilities in the country”.*

Senior NTP manager.

*“From what I saw in the data*, *I can say that*, *the private sector*, *kind of was*, *a “shock absorber” in quotes*. *So*, *I could see that they absorbed some shock from this COVID*. *But because …the private sector is still in the process [of becoming] optimum*, *… they absorbed as much [shock] as they could take”*.

NTP M&E staff.

### Availability of TB and COVID-related services

At the time of the survey in the second quarter of 2021 (Q2 2021), most surveyed facilities (69%) reported offering TB screening services (Table 2). The proportion of facilities offering TB screening services was highest (84% of 1,659) among SHOPS Plus-supported facilities, compared to 35% (out of 730) non-network facilities. These facilities also included 84% of clinics, as well as 62% of medicine vendors and 28% of pharmacies. Of facilities that did not conduct screening, 87% were pharmacies and medicine vendors, and most reported that they would refer patients elsewhere for testing.

**Table 2:** Response to survey questions with some state-level differences.

Questions	Responses	Kano 1,093 n(%)	Lagos 1,319 n(%)	Total 2,412 n(%)	p-value
Closures	Not closed	601 (55%)	1,075 (81%)	1,676 (69%)	< 0.001
	Closed once	465 (43%)	222 (17%)	687 (29%)	
	Closed more than once	27 (2%)	22 (2%)	32 (1%)	
* Reasons for closure	Government mandate/lockdown	458 (95%)	192 (87%)	650 (93%)	< 0.001
	Low client visits	4 (1%)	5 (2%)	9 (1%)	
	Not enough PPEs or other medical supplies	7 (2%)	2 (1%)	9 (1%)	
	Not enough staff available	1 (0%)	1 (1%)	2 (0%)	
	Citizen protests (EndSARS)	0 (0%)	7 (3%)	7 (1%)	
	Other reasons	10 (2%)	13 (6%)	23 (3%)	
Staff numbers	1 to 10	909 (83%)	1,018 (77%)	1,927 (80%)	< 0.001
	11 to 50	174 (16%)	271 (20%)	445 (18%)	
	51 to 100	10 (1%)	24 (2%)	34 (1%)	
	More than 100	0 (0%)	6 (1%)	6 (0%)	
Change in hours of operation since COVID	No change	785 (72%)	995 (75%)	1,780 (74%)	< 0.001
	Fewer hours	227 (21%)	294 (22%)	521 (21%)	
	More hours	81 (7%)	30 (2%)	111 (5%)	
Change in average number of clients per day	Yes	803 (74%)	1,014 (77%)	1,817 (75%)	< 0.001
	No	284 (26%)	269 (20%)	553 (23%)	
	Not sure	6 (0%)	36 (3%)	42 (2%)	
Compared to pre-COVID, how have client numbers changed?	Fewer patient visits per day	526 (48%)	803 (61%)	1,329 (55%)	N/A
	More patient visits per day	277 (25%)	211 (16%)	488 (20%)	
	Missing	290 (27%)	305 (23%)	595 (25%)	
Increased patient fees to cover the costs of PPE (e.g. gloves, masks, hand-sanitizer)	No	967 (88%)	1,198 (91%)	2,165 (90%)	0.034
	Yes	126 (12%)	121 (9%)	247 (10%)	
Change in consultation fees	No consultation fees	888 (81%)	1,048 (79%)	1,936 (80%)	0.279
	No change	181 (17%)	251 (19%)	432 (18%)	
	Increased	16 (1%)	14 (1%)	30 (1%)	
	Decreased	8 (1%)	6 (1%)	14 (1%)	
TB screening	Yes	788 (72%)	879 (67%)	1,667 (69%)	0.004
	No	305 (28%)	440 (33%)	745 (31%)	
COVID-related services offered	No COVID services	967 (89%)	969 (75%)	1,936 (81%)	
	COVID screening only	118 (11%)	304 (23%)	422 (18%)	
	COVID screening and testing	1 (0%)	9 (1%)	10 (1%)	< 0.001
	COVID screening and treatment	0 (0%)	10 (1%)	10 (1%)	
*Bi-directional screening	No screening	855 (78%)	929 (70%)	1,784 (74%)	< 0.001
	No	212 (20%)	277 (21%)	489 (20%)	
	Yes, everyone screened for TB is screened for COVID	26 (2%)	113 (9%)	139 (6%)	
Telemedicine use	No	885 (81%)	931 (71%)	1,816 (75%)	< 0.001
	Yes	208 (19%)	388 (29%)	596 (25%)	
Teleconsultation to identify, counsel or treat TB patients	No teleconsultations	885 (81%)	931 (71%)	1,816 (75%)	< 0.001
	No	117 (11%)	250 (19%)	367 (15%)	
	Yes	91 (8%)	138 (10%)	229 (10%)	
**Only clinical facilities (n = 628)**					
Changes in services offered	No change	147 (62%)	250 (64%)	397 (63%)	< 0.001
	Fewer services	56 (24%)	70 (18%)	126 (20%)	
	More services	25 (11%)	60 (15%)	85 (14%)	
	Both—some services stopped, and others added	10 (4%)	10 (3%)	20 (3%)	
Staff layoffs due to COVID	No	199 (84%)	352 (90%)	555 (88%)	< 0.001
	Yes	37 (16%)	35 (9%)	72 (11%)	
	Not sure	2 (1%)	3 (1%)	5 (1%)	
Shortages of medical supplies since COVID	No	199 (84%)	322 (83%)	521 (83%)	< 0.001
	Yes	39 (16%)	68 (17%)	107 (17%)	

*Questions with skipped pattern for affected facilities only.

N/A Chi-square invalid due to high proportion of missing data.

Most facilities (60%) reported that they collected samples on site and sent them to laboratories for analysis. Among laboratories, 141 of 258 (55%) reported the ability to process TB tests; another 30% (n = 78) reported that they collect samples for analysis at a different facility for TB testing.

**Fig 4:**
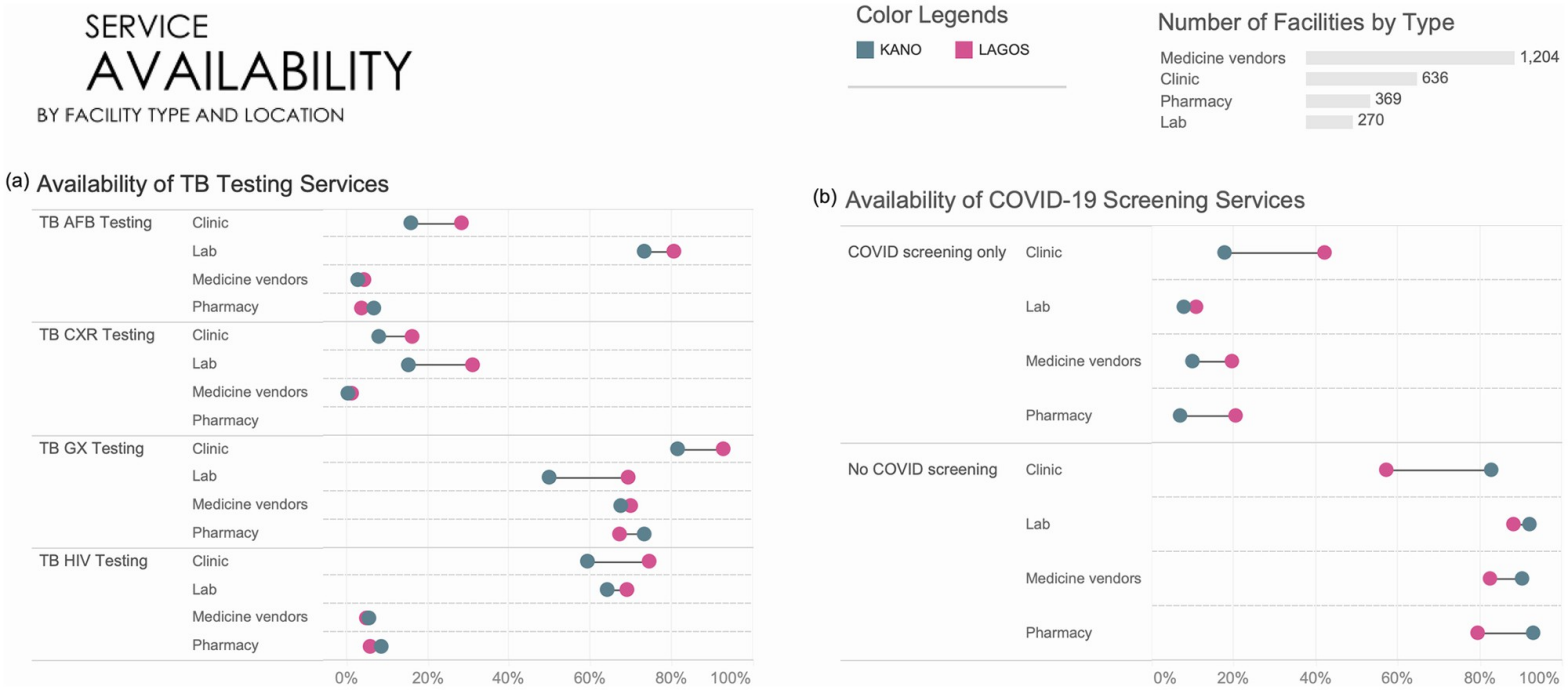
(a) Availability of TB screening services across facility type and location in Q2 2021; (b) COVID-19 screening services across facility type and location in Q2 2021.

Xpert MTB/RIF (Cepheid, Sunnyvale, CA), hereafter referred to as Xpert, was the most widely reported available test. Majorities of facilities of every type in both states reported that they were able to support Xpert testing for patients for TB. This included 88% of clinical facilities, 65%of pharmacies, 70% of medicine vendors and 60% of standalone laboratories. Fig 4a shows comparatively very low availability of AFB microscopy testing (except in laboratories), and chest X-ray facilities. Only clinical facilities and laboratories (65%) reported supporting HIV testing for TB patients.

Few facilities (17%, n = 422) reported making COVID-19 services available to their patients (Fig 4b), with 72% of these facilities (n = 304) located in Lagos State. Forty-three percent (43%) of clinical facilities in Kano reported that they supported screening, testing, or treatment of COVID, as did 17% of clinical facilities in Lagos. Availability of COVID testing was also similarly low among all other facility types. Fewer than 1% reported the availability of COVID-19 testing or treatment.

22% of facilities (521) reported being open fewer hours since the start of the COVID-19 pandemic, while 74% of facilities (1,780) reported that their hours had stayed the same. Most of the closures happened between March and May 2020, with only 5.6% of facilities (136) experiencing closures beyond that time. A majority (75%, 1,817) of facilities reported that their average clients seen per day had changed since the start of the pandemic, with 73% (1,329) reporting fewer patient visits per day. The OPD trend (Fig 3) supports providers’ verbal reports, as the May-July 2021 levels were not up to those seen during the same period in 2018, even though significant recovery had taken place.

### Short- and long-term COVID-19 disruptions on clinical facilities and adaptations

**Fig 5:**
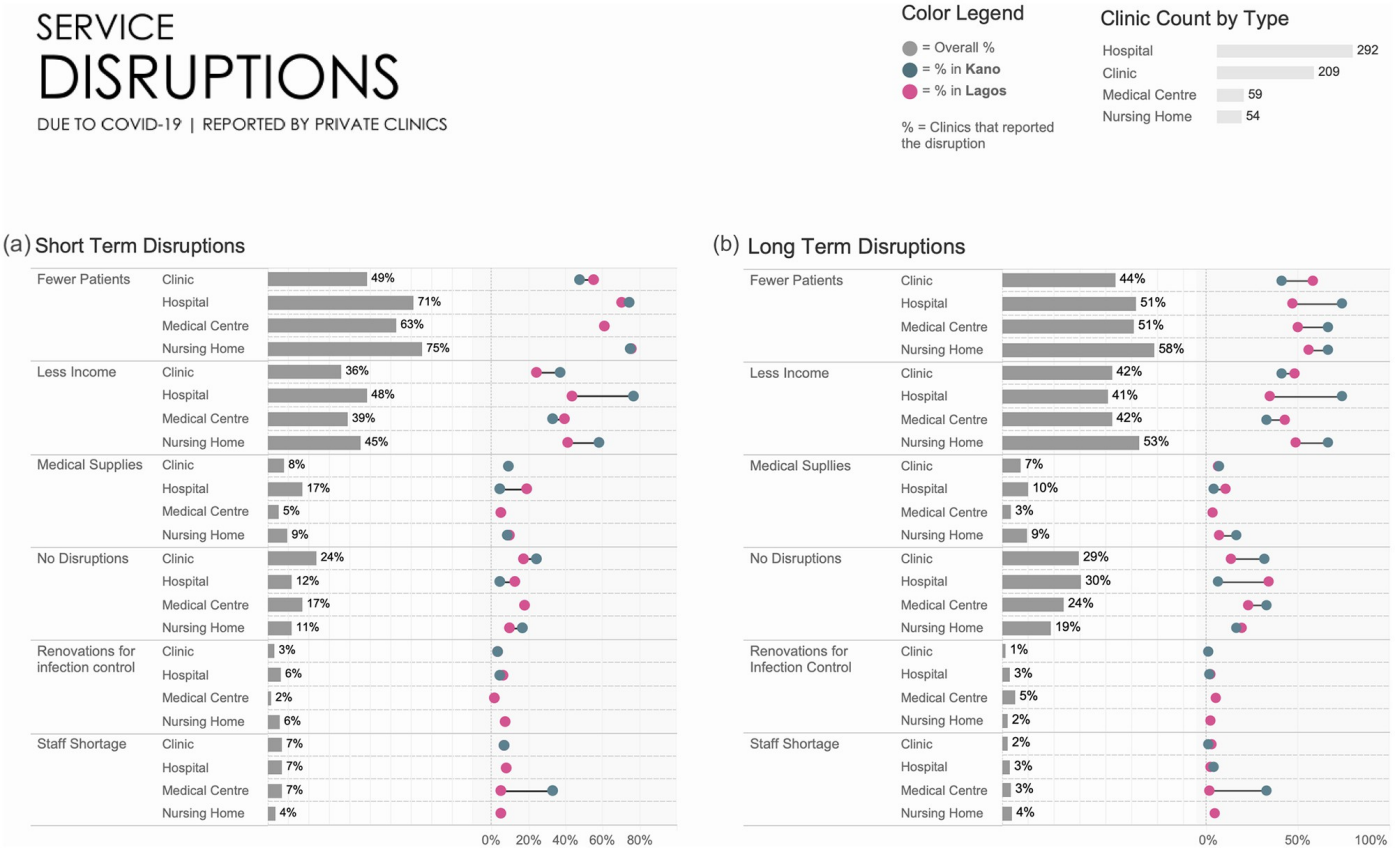
(a) Short- term disruptions due to COVID-19 reported by private clinics in Kano and Lagos; (b) Long-term disruptions due to COVID-19 reported by private clinics in Kano and Lagos.

Private clinical facilities reported both short- and long-term disruptions due to COVID-19 on availability of medical supplies, staff, required renovations for infection control, patient volume and income (Fig 5). Providers were asked about disruptions in their practice between March 2020 and Q2 2021. We assessed both short-term (1–3 months in duration) and long-term (above three months) disruptions. Short-term decreases in caseload were reported by 48% of clinics, 71% of hospitals, 63% of medical centres, and 74% of nursing homes (Fig 5a). In comparison, long-term decreases in caseload were reported by 44% of clinics, 51% of hospitals, 51% of medical centres, and 57% of nursing homes (Fig 5b). Simultaneously, 35% to 48% of facilities reported that their income has reduced.

Themes from the interviews also showed that income was reduced, particularly for smaller private facilities.

*“The pandemic with the attendant lockdown, restriction of movement, affected mostly the smaller hospitals that are struggling, so most of them were not having the clients, and so it affected drastically, the income of most private hospitals.”*

Senior NTP staff.

**Fig 6:**
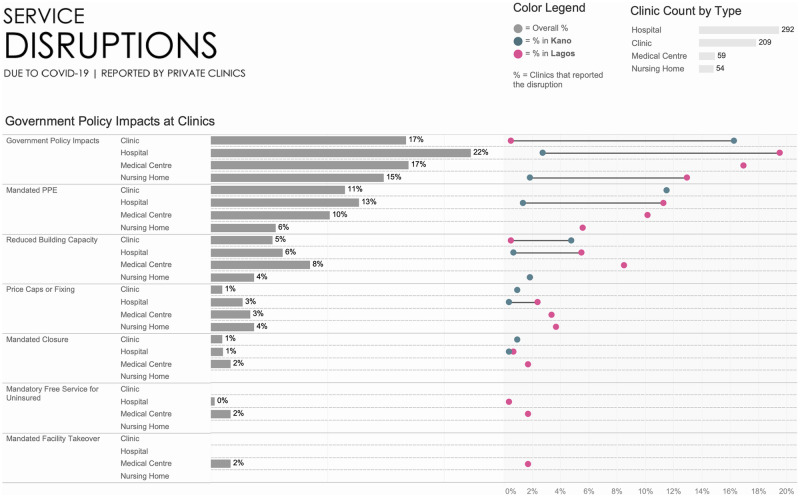
Service disruptions due to COVID-19.

Overall, few facilities indicated being impacted by governmental policies implemented during COVID-19 (Fig 6). Of these policies, the mandated use of PPEs had the biggest impact, followed by social distancing rules.

Some policymaker interviews indicated that the government COVID-related policies worked at the beginning, but there were later problems with compliance and enforcement.

*“We have set out some basic, you know, regulatory notes and also protocols to be followed… but unfortunately some private health facilities violated … rights that [were] given to them temporarily.., they violated [guidelines] and they were forced to close down…”*

Senior MOH staff

### Changes to costs of service during the COVID-19 pandemic

10% of surveyed facilities (n = 247) reported increases to patient fees to cover additional PPE costs. Of these 247 facilities, 49% (n = 120) reported increasing the price of medications, 42% (n = 104) reported adding a separate PPE fee on patients’ bills, and 13% (n = 32) increased the prices of laboratory fees. Compared to pre-COVID times, most surveyed facilities reported that their registration fees (93%), consultation fees (91%) or GeneXpert test prices (99%) were unchanged.

### State-level differences to the impacts of COVID-19

The two states in our study differed in multiple ways and in their experience with COVID-19. The descriptive statistics and Pearson chi-square reflecting some of these differences are shown in Table 2. There were statistically significant associations between the States and closures during the lockdown period, staff numbers, change in hours of operation, client load, and service provision for TB and COVID, as well as telemedicine use.

A higher proportion of facilities in Kano (492 facilities or 45% compared to 244 facilities or 18%) reported having closed at one or more times due to COVID-19 control measures, compared to facilities in Lagos (18%, n = 244 facilities).

Several State-level differences are highlighted in our figures. The TB notifications chart (Fig 3) shows that while OPD numbers were consistently higher in Lagos, the numbers and post-lockdown recovery were generally stronger in Kano for total screened, total presumptive and total diagnosed.

Fig 4a shows that overall, a majority of providers, most of whom received support from SHOPS Plus, were providing TB services in Q2 2021. A smaller percentage of clinical facilities in Kano (78%) compared to Lagos (92%) offered TB screening, while more medicine vendors in Kano (73%) compared to Lagos (55%) offered TB screening. AFB testing in clinics were lower in Kano (16%) than in Lagos (28%) and chest x-rays were lower in clinics (8% vs 16%) and laboratories (15% vs 31%) in Kano than in Lagos. Xpert availability also differed between the states– 93% in Kano compared to 82% of clinics in Lagos, and 69% vs 50% in laboratories. There was similar availability in pharmacies and medicine vendors.

COVID screening *(Fig 4b)* differed significantly between Kano and Lagos for clinics (18% vs 42%), pharmacies (20% vs 7%) and medicine vendors (19% vs 10%).

For disruptions lasting 1–3 months (Fig 5a), some of the major differences included lower income reported by 77% of providers in Kano hospitals compared to 43% of providers in Lagos; shortage in medical supplies in 5% of Kano and 19% of Lagos hospitals; and staff shortages in 33% of Kano and 5% Lagos medical centres.

For longer disruptions (3–6 months, Fig 5b), there were several noticeable differences: more patients were reported for hospitals medical centres and nursing homes in Kano than in Lagos; lower income in 74% of Kano compared to 35% of Lagos hospitals; and staff shortages in 33% of Kano compared to 2% of Lagos medical centres.

There were also variations on the impact of Government policies across facility types in the two States for overall impact, PPE use, and social distancing but less so for price caps and mandated closures *(Fig 6)*. For example, 1% of hospitals in Kano compared to 11% of hospitals in Lagos mandated PPE use, and 1% in Kano vs 5% in Lagos of facilities limiting number of clients within the premises.

### NTP support measures and provider adaptations to COVID-19

From the interview data, policymakers revealed that the National program, in collaboration with stakeholders like the SHOPS Plus, put in place several measures to support providers in service delivery. For example, TB commodity and drug supplies were increased to ensure facilities had enough despite the movement restrictions. Medication refill protocols were adapted to allow for patients being referred from public facilities to private facilities if those public facilities ran out of TB medications. Additionally, TB treatment facilities were required to give patients up to a month’s supply of TB medications, rather than the usual two-week supply.

*“The first thing was that, we had to quickly change our policy on supply of commodities. So, we had to give more than… We usually have a calculation of the expected number of drugs or consumables that the facility can use at a particular period of time to avoid expiries, but we quickly had to change that because we didn’t know how long the restriction was going to last. So, distribution of drugs, commodities to the facilities changed and the number of months they were given. Also, we changed our policy on patient management and we immediately rolled out a document to all facilities to ensure that all patients are given drugs for up to a month.”*

Senior NTP staff.

*“To mitigate against [COVID disruptions] we ensured that some of our facilities, especially the… we gave instructions from the State TB Control program… that [facilities] should give at least 1 month stock of drugs to patients enrolled in their facilities, so that we won’t have the issue of stock-out of drugs, such that the adherence to treatment is maintained by our patients, especially those in the private facilities as well as the public facilities.”*

MOH TB staff, Kano State.

The NTP, in collaboration with states, increased contact tracing and encouraged home-based care.

*“We had more cases than we can handle, that necessitated the proactiveness in [establishing] home-based care program, where following some basic sets of criteria, individuals … treated at home, they are overseen by some of our healthcare workers, either through phone calls or directly, physically, visiting their homes.”*

Senior MOH staff, Kano State.

The states also put in additional measures of their own. The Kano STBLCP gathered key private providers, mentored them, and gave them guidance to mitigate the impact of COVID-19 on TB detection. They engaged PPMVs, which community members often visit first for services, to provide TB screening for clients. Roaming screeners were used, which was an innovation by SHOPS Plus, to go throughout the community to make sure all presumptive TB patients, who may have been disinclined to go to clinics, were connected to testing in nearby laboratories.

*“We provided engagement and mentorship to the private providers… strengthened actually by partners in the private sector, SHOPS Plus, where we gathered some of the key private providers, mentored them, talked them through, give them guidance, and mentored to mitigate the impact of COVID-19, on what strategies can we do… And then even roaming screeners… that came up as innovation through our support of private partner… These roaming screeners, also go community to community, street by street, you know, area by area, to make sure that all our presumptive TB cases, who may not be able to present themselves before our clinics, are been attended to in the communities,.. with PMV engagement and the nearby laboratories and then the support of the EQA (external quality assurance) team, they are able to have their samples collected and … screened for TB.”*

Senior MOH staff, Kano State.

Policymakers also mentioned that the government collaboration with implementation partners like SHOPS Plus, helped to maintain TB case finding. Concerted efforts were also required through coordination of various governmental agencies with the private sector. One policymaker also expressed how the favourable response from the private sector has been a key factor in addressing the pandemic:

*“Some [private facilities] have even gone ahead to procure some equipment that protect the hospital on their own not through support, some have also gone ahead to even provide testing facilities for government to utilize. So, they have also played an important role in the area of trying to support government to address COVID issues, from their own perspective. So, for me, I think their response have been so nice, because they are buying equipment for COVID, nobody is paying them for that. They’re not even charging so much for those areas. They have also given current opportunity to use those services for when government become overwhelmed by their own system, they also provide.”*

Senior NTP TB staff.

## Discussion

Our study to assess changes to private sector practices interviewed policymakers and surveyed providers in two large Nigerian cities after the second COVID-19 wave. Our findings show that at the provider level, there were short-term disruptions, particularly wide-spread closures seen during the lockdown periods. Most facilities of all types reported short-term closures due to COVID-19, and closures were primarily due to the mandatory lockdowns. However, frequency and duration of closures was much less among pharmacies and laboratories, and after January 2021, very few providers reported spending time out of service.

However, facilities, most of which were intensively engaged by the SHOPS Plus program, had recovered very largely and their capacity to screen and manage TB patients were affected minorly. Within the group of providers we surveyed, TB screening and testing services had largely recovered from the impact of the pandemic at the time of the survey in May and June 2021, as around 70% of all providers reported that TB screening was available at their facilities, with others indicating that they regularly referred clients to other sites for TB diagnostics. This was expected as most of the providers (within the SHOPS Plus network) were actively supported to provide screening at the time of the survey. GeneXpert was the most widely reported available test, indicating that the recommended diagnostic has been scaled up in private facilities in these two states. Our findings differ from earlier reports of massive disruptions in TB screening due to the use of GeneXpert for COVID screening reported in several countries [3,17,26], although this might be due to the limited availability of the GeneXpert COVID cartridges [27]. Many authors have called for an integrated and phased approach to the use of GeneXpert for COVID screening in order to minimise disruptions on TB/HIV services in weak, low resource public health systems [1,28–30].

Few providers reported that they offered screening for COVID-19. These findings support reports indicating that only public facilities were given the mandate to offer COVID-19 testing and treatment at the beginning of the pandemic [13,20]. Our findings also seems to suggest limited availability of COVID-19 screening and testing, especially at private clinical facilities, despite the Federal Ministry of Health’s announcement that COVID-19 testing would be scaled up to eligible private hospitals [20].

Similarly, most providers reported seeing fewer patients and earning less income.

Providers said fewer patients were showing up in the private facilities, even though most of their facilities remained open during the lockdown periods, leading to decreased provider income. Participants mentioned other reasons for patients avoiding facilities, including transportation and movement restrictions. At the time of the survey, however, provider TB case notifications had bounced back to pre-pandemic levels.

Given that the private sector was perceived to be able to “pick up some of the slack” from the public sector (which was burdened with the bulk of the COVID-19 response), some policymakers remarked that they were more aware than ever of the importance of collaboration and coordination between government stakeholders and the private sector. They called for increased efforts to shape and encourage private sector engagement. According to the NTP policymakers, the government is also developing a policy that would formally encourage the engagement of private health facilities in the prevention, treatment and care of tuberculosis in Nigeria.

Other detailed reports of facility closures, service disruptions, and increases in costs for patients have begun to be published [15,16,31,32]. The results of our study agree with results obtained in a previous rapid assessment by Klinton et al. on the impact of the pandemic on TB services in the private sector in seven high-burden TB countries including Nigeria [16]. This rapid assessment reported fear of COVID-19 infection in facilities on both the patient and provider side, which was heightened by the overlapping TB and COVID-19 symptoms. The authors also mentioned financial constraints faced by the private sector and increased patient costs, which are also reflected in our data for clinics and laboratories. Additionally, our data did not reflect a significant uptake in the use of teleconsultations in the private sector in Kano and Lagos. This contradicts a recent cross-sectional survey of private and public healthcare providers and consumers in Nigeria reporting that 63% of consumers received telemedicine services during the pandemic [33]. Several studies have highlighted the potential telemedicine offers in increasing healthcare access in low and middle income countries, particularly since the onset of the pandemic [34,35], even though its usage might be more suited to high-income settings with adequate access to smart phones, internet connectivity and constant electricity [17,36]. Intersectoral collaboration incorporating the private sector has also been highlighted as an important element of pandemic outside a TB context in Colombia [37], which indicates that PPM could benefit management of various diseases, not only TB.

For nearly three decades, private sector engagement has been recommended in the fight against TB as an effective strategy to tackle low detection [9,38–40]. Strengthening TB/HIV service delivery was one of goals of the National Public Private Partnership for Health policy developed by the Nigerian government. In 2005 [41,42]. Despite this, public-private mix (PPM) strategies have had limited uptake. In Nigeria, private providers account for 67% of initial healthcare-seeking, yet only account for 12% of TB case notification in the country [9,10]. One barrier to effective private provider engagement might be the lack of a consolidated database of all private providers in the country. While the government of Nigeria maintains a list of registered private health facilities [25], it only lists clinical facilities, and it is unclear how often this list is updated. This highlights the need for further engagement of this sector. Given the recent uptick of TB deaths and decrease in TB notifications as well as reports that the pandemic has resulted in a global setback of 12 years’ worth of progress on TB elimination [2,25,43], it is important to double-down on public-private partnerships.

This study has several limitations. The number of facilities that may have closed permanently in March 2020 due to COVID-19 could not be assessed as facilities that were permanently closed and no longer reachable by SHOPS Plus staff were excluded from this survey (11.4% of all facilities that had ever reported data within the network), a potential source of selection bias. Additionally, as most sampled providers were members of the SHOPS Plus network and have been sensitized to increase the provision of TB services, the results detailed here are not generalizable to all similar urban settings in Nigeria or to other providers in those cities. Additionally, this study was conducted only in Kano and Lagos, Nigeria’s biggest cities, limiting its generalizability to smaller and more remote locations in Nigeria. It would be important to expand the scope of this research to further understand the impact of the pandemic during 2022 and adaptations in other states. However, this study provides a detailed insight into facility closures and service interruptions among private providers in Lagos and Kano state that is to date not explored in any other published article.

## Conclusion

The COVID-19 pandemic has had devastating effects throughout the globe, and its strongest impact has been towards the most vulnerable. TB and COVID-19 both being respiratory diseases presenting with similar symptoms results in unique challenges for the management of TB. This has resulted in significant setbacks in TB elimination targets and important challenges in accessing services for those living with TB. The private sector plays an important role in the provision of healthcare services in Nigeria, and thus cannot be overlooked in TB programming. The results from this study highlight the important role private providers can play in supporting both COVID-19 and TB, despite facing important financial hurdles due to the pandemic, and that private sector engagement is an important driver in overall health system strengthening in the country.

## Supporting information

S1 Fig Sampling frame and survey response. Constructing the enumeration lists for the COVET Facility Survey in Nigeria frame was a multi-step process conducted using data collected through September 2020. Eligible facilities were operational and had known location information. SHOPS Plus network facilities were identified using the SHOPS Plus monthly program monitoring dataset containing service delivery data submitted by network facilities between 2018 and September 2020. There were 2,405 unique facilities that had ever reported data as a SHOPS Plus network member. Of those, 275 facilities were determined by SHOPS Plus program staff to be closed or unknown operational status as of September 2020 and were excluded from the study. We were unable to determine what proportion of these closed as a result of COVID-19 or other reasons. 234 facilities had no location information available and were considered ineligible for this study, leaving 1,896 SHOPS Plus network facilities included in the sampling frame. An additional 1,007 non-network heath facilities were included in the enumeration list, identified from a dataset of facilities that were assessed in 2018 to gauge interest and suitability to participate in a SHOPS Plus program network. Facility names from this database were cross-referenced with the list of unique SHOPS Plus network facilities. Any facilities in the 2018 Assessment dataset that did not have a match on name in the SHOPS program data list were assumed to be eligible for this study as non-network facilities. Data collectors tried to increase overall percent of target achievement by attempting to survey additional SHOPS Plus or non-network facilities that did not make it on to the original enumeration list (i.e., because these facilities lacked minimal contact/location information), but because so little information was available for these facilities these back-up lists yielded very few additional successful interviews. Twenty-three additional facilities (15 in Kano, 8 in Lagos) of unknown network status are included in the total.
(TIF)
