# Supplementary figures and images for: Tuberculosis service disruptions and adaptations during the first year of the COVID-19 pandemic in the private health sector of two urban settings in Nigeria—A mixed methods study
Source: PLOS Glob Public Health. 2023 Mar 24;3(3):e0001618. doi: 10.1371/journal.pgph.0001618 (PMC10038269; doi:10.1371/journal.pgph.0001618)

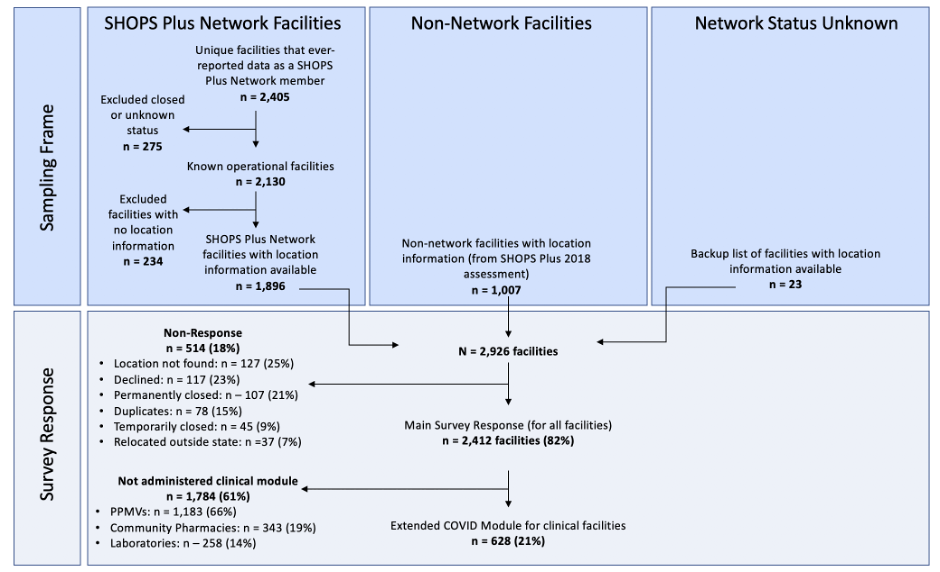

Supplement: S1 Fig — Constructing the enumeration lists for the COVET Facility Survey in Nigeria frame was a multi-step process conducted using data collected through September 2020. Eligible facilities were operational and had known location information. SHOPS Plus network facilities were identified using the SHOPS Plus monthly program monitoring dataset containing service delivery data submitted by network facilities between 2018 and September 2020. There were 2,405 unique facilities that had ever reported data as a SHOPS Plus network member. Of those, 275 facilities were determined by SHOPS Plus program staff to be closed or unknown operational status as of September 2020 and were excluded from the study. We were unable to determine what proportion of these closed as a result of COVID-19 or other reasons. 234 facilities had no location information available and were considered ineligible for this study, leaving 1,896 SHOPS Plus network facilities included in the sampling frame. An additional 1,007 non-network heath facilities were included in the enumeration list, identified from a dataset of facilities that were assessed in 2018 to gauge interest and suitability to participate in a SHOPS Plus program network. Facility names from this database were cross-referenced with the list of unique SHOPS Plus network facilities. Any facilities in the 2018 Assessment dataset that did not have a match on name in the SHOPS program data list were assumed to be eligible for this study as non-network facilities. Data collectors tried to increase overall percent of target achievement by attempting to survey additional SHOPS Plus or non-network facilities that did not make it on to the original enumeration list (i.e., because these facilities lacked minimal contact/location information), but because so little information was available for these facilities these back-up lists yielded very few additional successful interviews. Twenty-three additional facilities (15 in Kano, 8 in Lagos) of unknown networ [file pgph.0001618.s001.tif]
